# Smart Interactive Education System Based on Wearable Devices

**DOI:** 10.3390/s19153260

**Published:** 2019-07-24

**Authors:** Jia-Ming Liang, Wei-Cheng Su, Yu-Lin Chen, Shih-Lin Wu, Jen-Jee Chen

**Affiliations:** 1 Department of Computer Science and Information Engineering, Chang Gung University, Taoyuan 33302, Taiwan; 2 Department of General Medicine, Chang Gung Memorial Hospital, Taoyuan 33378, Taiwan; 3 Department of AI Innovation Research Center, Chang Gung University, Taoyuan 33302, Taiwan; 4 Department of Cardiology, Chang Gung Memorial Hospital, Taoyuan 33305, Taiwan; 5 Department of Electrical Engineering, Ming Chi University of Technology, New Taipei City 24301, Taiwan; 6 Department of Electrical Engineering, National University of Tainan, Tainan 70005, Taiwan; 7 College of Artificial Intelligence, National Chiao Tung University, Tainan 71150, Taiwan

**Keywords:** intelligent interactivity, instant feedback, wearable device, data analysis, learning concentration, education system

## Abstract

Due to the popularity of smart devices, traditional one-way teaching methods might not deeply attract school students’ attention, especially for the junior high school students, elementary school students, or even younger students, which is a critical issue for educators. Therefore, we develop an *intelligent interactive education system*, which leverages wearable devices (smart watches) to accurately capture hand gestures of school students and respond instantly to teachers so as to increase the interaction and attraction of school students in class. In addition, through multiple physical information of school students from the smart watch, it can find out the crux points of the learning process according to the deep data analysis. In this way, it can provide teachers to make instant adjustments and suggest school students to achieve multi-learning and innovative thinking. The system is mainly composed of three components: (1) smart interactive watch; (2) teacher-side smart application (App); and (3) cloud-based analysis system. Specifically, the smart interactive watch is responsible for detecting the physical information and interaction results of school students, and then giving feedback to the teachers. The teacher-side app will provide real-time learning suggestions to adjust the teaching pace to avoid learning disability. The cloud-based analysis system provides intelligent learning advices, academic performance prediction and anomaly learning detection. Through field trials, our system has been verified that can potentially enhance teaching and learning processes for both educators and school students.

## 1. Introduction

Smart devices have brought tremendous changes to human life, especially for the young people. Traditional teaching skills might be unable to attract school students’ attention nowadays and a new intelligent teaching system is required for the modern society. According to this, we investigate a *smart interactive educational learning system* based on wearable devices, which integrates smart watches with a smartphone application (App) that can interact with school students and educators, and make teaching and learning processes more interesting and concentrated. This system leverages the machine learning technique and data analysis based on school students’ interaction data, physical information, and learning performance, and then models them as the learning benefit relevance. In this way, the teacher can easily control the learning situation in the classroom and also can adjust the teaching contents appropriately in time to prevent school students from distracted in the class. Specifically, the system can predict the trend of learning performance by analyzing the interaction feedback, physical information, and test scores, and provide suitable learning suggestions to improve teaching and learning efficiency.

The major contributions of this paper are three-fold. First, this is the first work to integrate with smart watch, smartphone, and the could-based service to enhance the teaching and learning processes for both educators and students. Second, the system is designed based on wearable sensing technologies and machine learning techniques so as to provide interesting interactive functions, including: (1) random grouping; (2) hand-gesture answer; (3) group competition; (4) instant questioning; (5) automatic roll call; and (6) analysis and prediction of learning performance, which can make students more concentrated in class and enjoy learning. Third, through the field trials, our system has been validated at Taoyuan Municipal JieShou Junior High School, Taiwan.

## 2. Related Work

In the literature, several researches have studied the classroom interactive feedback systems [1,2,3,4,5,6,7,8,9,10,11]. The work [1] explores the influence of the traditional push-button voting system on learning concentration. It points out that the interactive feedback system can help to increase school students’ concentration; however, it neglects the features to inspire students. In [2,3], the authors design a smartphone application to attract students’ attention; however, they may distract students by other gaming applications from smartphones. In [4], the authors use short messages on mobile phones to increase the interactivity in class, but it may not prevent students from repeatedly asking the same questions and discussing other things irrelevant to the class. In [5], the authors propose using a ‘clicker’ device in the classroom to improve the interaction between teachers and students. However, this device only can record the students’ attendance and answer simple questions; thus, it cannot provide better interactive functions such as hand-gesture answer, random grouping, group competition, analysis, and prediction of learning performance. In [6], the authors proposes a web-based classroom response system to promote active learning. However, it may not prevent students from playing other web games in the classroom. In [7,8], the authors propose a game-based student response system (SRS) to improve students’ participation; however, these games may also lead to distraction from learning. From [9], the authors show that the exercise and concentration are highly correlated. In [10,11], the authors mention that the wearable devices are emerging to enhance learning efficiency. Therefore, based on the above observations, it motivates us to investigate a smart interactive education system with wearable devices to achieve an innovative educating and learning.

## 3. System Design

**Figure 1:**
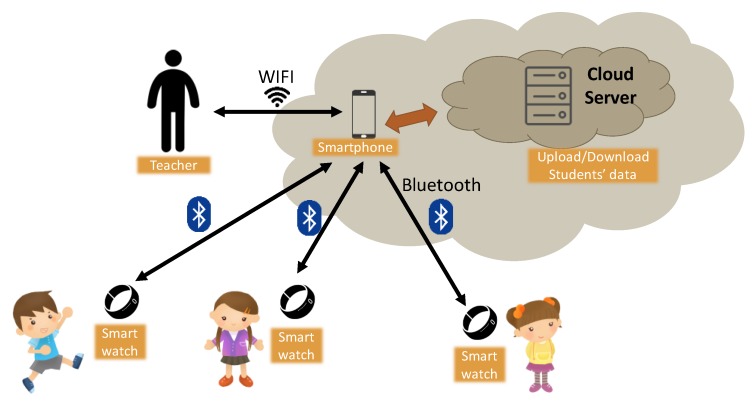
System architecture.

Figure 1 shows the architecture of our system, which consists of three components: (1) smart interactive watch; (2) teacher-side smart app; and (3) cloud-based analysis system.

**Figure 2:**
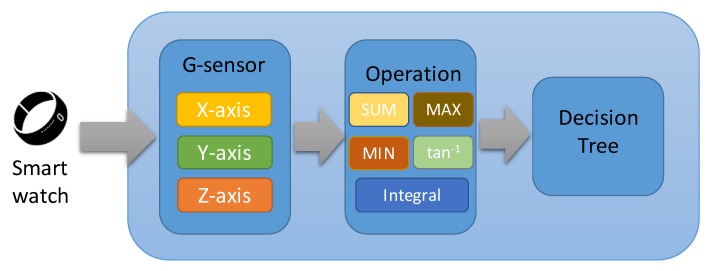
Flowchart of hand-gesture recognition.

1. **Smart Interactive Watch**: to collect school students’ learning interactions and physical information, where learning interactions include the number of times for raising hands and answering questions, and the corresponding response time in personal/group competitions; physical information includes heart rate, exercise strength (number of walking steps), activity frequency, etc. It is also responsible to report such information to the teacher-side application and cloud-based system for further analyses.
2. **Teacher-side Smart App**: to receive the interactive information from the smart watch and provide special functions for teaching so that the teacher can immediately understand school students’ learning status in the classroom. The specific functions are as follows:
  - *Random grouping*: school students will be informed and divided into the specific number of groups according to the teacher’s setting, such as the group size, gender, or learning performance, etc. This can save lots of class time and avoid the unfair doubts.
  - *Hand-gesture answer*: to recognize the answer of the yes-no question through the hand-gesture of ‘○’ and ‘╳’ from smart watches (by moving hands in a circle and a cross motion, respectively) and detect the answer of the multiple-choice question through the hand-gesture of ‘↑’, ‘↓’, ‘←’, and ‘→’ by waving hands in up, down, left, and right directions. After receiving the answers from smart watches through Bluetooth communication, this app will check the results automatically and send the feedback to the smart watches and then perform vibration. Note that the hand-gesture activities are recognized based on the decision tree scheme, which is a machine learning model, by referring the values of three-axis accelerometer from the smart watch. The flowchart of hand-gesture recognition is shown in Figure 2.
  - *Group competition*: after grouping, the teachers can obtain the interaction results and rank of their scores to achieve group competition.
  - *Instant questioning*: school students can ask questions quietly and privately by clicking on the smart watch, which is more suitable for introverted school students.
  - *Automatic roll call*: through the connection between the smart watch, the roll call can be realized automatically to reduce the waste of time in class.
3. **Cloud-based Analysis System**: through the collected data from smart watches and teacher-side app, the cloud system can deeply analyze and predict the learning performance based on the machine learning technology by referring the results of school students’ answer, group competition, instant feedback, physical information, and then conduct prediction for future learning scores. In addition, the system can provide learning advices and teaching suggestions for school students and teachers, respectively. Specifically, we leverage two prediction methods with the analysis regression of score prediction, called Linear Regression (LR) and Local Weights Linear Regression (LWLR) [12]. The main difference between the two prediction methods is that LR calculates only a single weight value for all the school students in class and LWLR finds the weight value individually that matches each student’s result to achieve an accurate score prediction. In addition, each established model will adjust the weights after a period of time to make the predicted score interval more realistic. The details of the prediction method are described as follows:

• **Method I: Linear Regression (LR)**

For the collect information from class interaction, it calculates the test scores, the overall class situation, and weight of the all class, and then takes the weight value into the following functions and constructs a model:

(1) $$y=\theta_{0}+\theta_{1}X_{1}+\theta_{2}X_{2}+\theta_{3}X_{3}+\theta_{4}X_{4}$$

(2) $$\{\begin{array}{c} \sum y=n\theta_{0}+\theta_{1}\sum x_{1}+\theta_{2}\sum x_{2}+\theta_{3}\sum x_{3}+\theta_{4}\sum x_{4}\\ \sum x_{1}y=\theta_{0}\sum x_{1}+\theta_{1}\sum x_{1}^{2}+\theta_{2}\sum x_{1}x_{2}+\theta_{3}\sum x_{1}x_{3}+\theta_{4}\sum x_{1}x_{4}\\ \sum x_{2}y=\theta_{0}\sum x_{2}+\theta_{1}\sum x_{1}x_{2}+\theta_{2}\sum x_{2}^{2}+\theta_{3}\sum x_{2}x_{3}+\theta_{4}\sum x_{2}x_{4}\\ \sum x_{3}y=\theta_{0}\sum x_{3}+\theta_{1}\sum x_{1}x_{3}+\theta_{2}\sum x_{2}x_{3}+\theta_{3}\sum x_{3}^{2}+\theta_{4}\sum x_{3}x_{4}\\ \sum x_{4}y=\theta_{0}\sum x_{4}+\theta_{1}\sum x_{1}x_{4}+\theta_{2}\sum x_{2}x_{4}+\theta_{3}\sum x_{3}x_{4}+\theta_{4}\sum x_{4}^{2} \end{array}$$

(3) $$\hat{\theta }={\left(X^{T}X\right)}^{-1}X^{T}Y$$

where *y* is the test score, *x*_1_ is the instant question score, *x*_2_ is the hand-gesture answer, *x*_3_ is group competition result, and *x*_4_ is the amount of personal exercises. This can calculate the most suitable coefficients in the class. Finally, by using the matrix operation in Equation (2), the weight values are calculated as a regression model.

• **Method 2: Local Weights Linear Regression (LWLR)**

When calculating the predicted scores, unreasonable score predictions may occur when the general performance of the all class is very good or very bad, leading to difficulty in detecting the situation that some school students are more prominent or whose performance needs improvement. In order to reduce the inaccuracy of these predictions, we use the local weighted linear regression to give each student their own weights; then, more accurate prediction of future scores will be:

(4) $$J\left(\theta \right)=\Sigma_{i}w^{\left(i\right)}{\left(y^{\left(i\right)}-\theta^{T}x^{\left(i\right)}\right)}^{2}$$

(5) $$w^{\left(i\right)}=\exp\left(-\frac{{\left(\chi^{\left(i\right)}-x\right)}^{2}}{2k^{2}}\right)$$

and:

(6) $$\hat{\theta }={\left(X^{T}WX\right)}^{-1}X^{T}WY$$

where Equation (4) means LR plus the respective weight which yields the regression result of LWLR. Equation (5) is the exponential decay function, which can adjust the value of *k* to find the best predicted model. Finally, through the matrix operation in Equation (6), it can find the coefficients that fit each student and then construct an individual prediction score model.

## 4. System Implementation and Performance Verification

### 4.1. System Implementation

**Figure 3:**
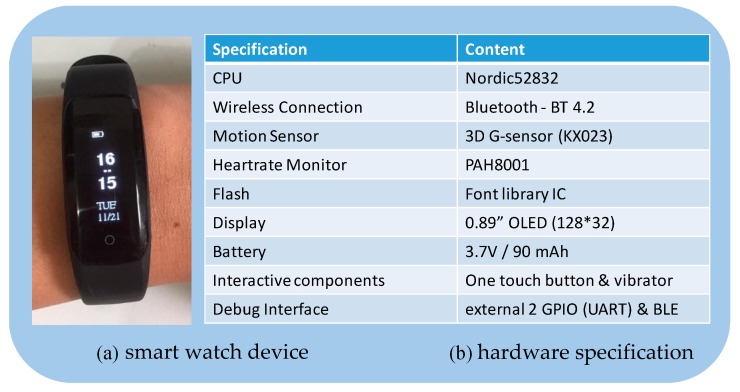
The specification of the Alfa Bracelet DS62.

The system has been implemented. The hardware specifications are listed as follows. The smart watch is developed based on Alfa Bracelet DS62 (produced by Alfaloop Inc., Taiwan, as shown in Figure 3a), where the operating system is Alfa OS v1.0. Note that the wireless module is Bluetooth 4.0, which is used for connecting with the teacher-side smart app. The smart app is developed based on Android 6.0.1 and operates on the smartphone (model: ze550kl, produced by ASUSTeK Computer Inc., Taiwan, with Qualcomm S615, 2 GB RAM and 16 GB ROM). The cloud system operates on the platform of Intel Core i5-6400 CPU 2.70 GHz, DDR3-1600 4 GB SDRAM, 1 TB HDD, and the operating system is Windows 10 Pro (64-bit), where the database is MariaDB v10.

### 4.2. Performance Verification

The following results show the field experiments at Taoyuan Municipal JieShou Junior High School, Taiwan. The total number of students was 18. In the classroom, the teacher divided all the students into six groups through the function of random grouping, where each group has three students. Then, the teacher asked ten questions by the function of instant questioning and five questions were performed by the group competition. Finally, three tests were completed by hand-gesture answering.

**Figure 4:**
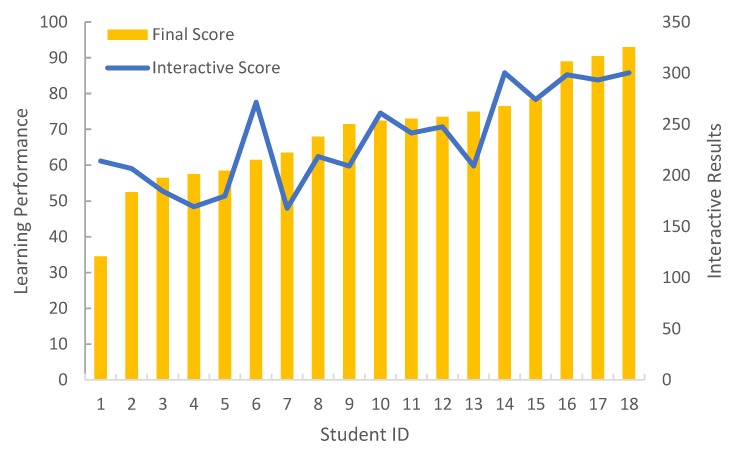
Comparison on the impact of interactive results on learning performance.

For the verification of learning process, we conduct the experiment to verify the impact of the students’ interactive results from the proposed system on the learning performance, where the interactive results are calculated based on the hand-gesture answer, group competition, instant questioning, etc., collected by the proposed system. In Figure 4, we can see that most of the students’ learning performances are positively correlated to the interactive results. This is because our system can attract students’ attention, making them concentrate on the teaching content and enjoy the learning process.

**Figure 5:**
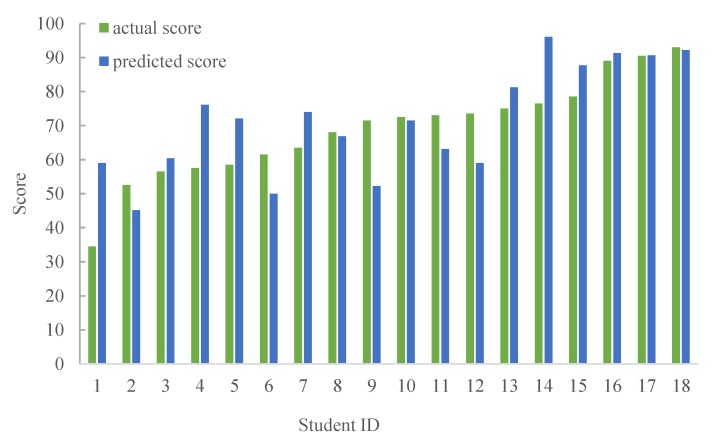
Comparison on predicted score and actual score of LR.

**Figure 6:**
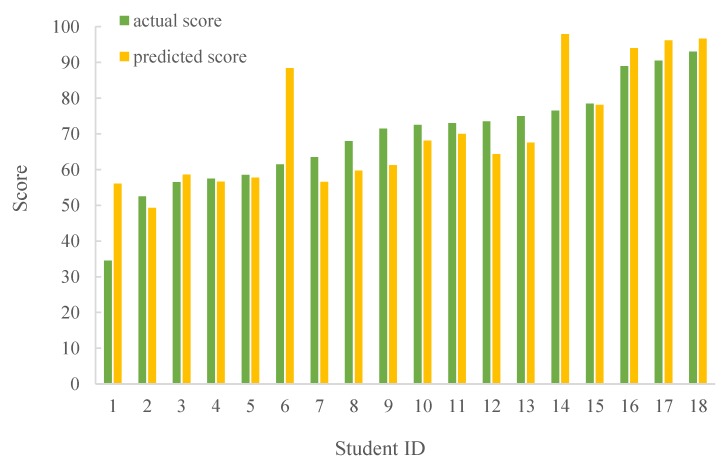
Comparison on predicted score and actual score of LWLR.

**Figure 7:**
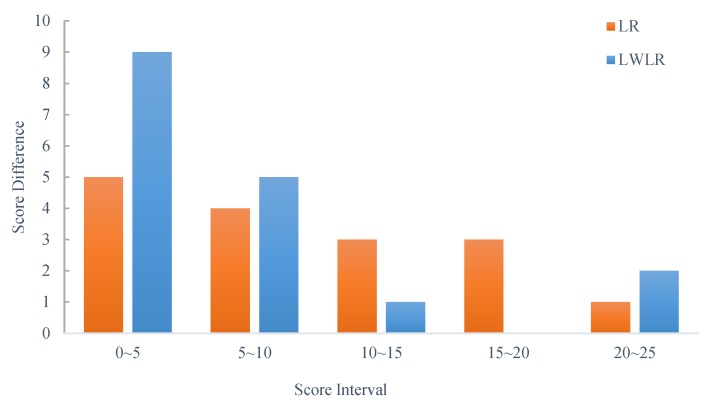
The results of predicted score differences between LR and LWLR.

For the analysis and prediction of school students’ grades, Figure 5 and Figure 6 show the results between the predicted and actual scores by LR and LWLR, respectively. Note that we use multiple scores in the classroom, including the results of personal/group answers, hand-gesture results, group competitions, and amount of exercises, test scores, and exam scores, to construct the models, where 2/3 of the data is used for the modeling and 1/3 of the data is used for the testing. Then, after exchanging the test data and modeling data, two more accurate models are obtained. Finally, parts of the scores in the course are applied for the model to obtain the predicted scores. As can be seen, the average difference between the predicted score and actual score is less than 9.6%, implying that the performance results are fairly consistent. In addition, Figure 7 shows the results of different scores intervals on the predicted score differences by LR and LWLR. We can see that the predicted score of the interval 0~5 is more accurate for both LR and LWLR methods. 

## 5. Conclusions

In this paper, we have introduced a smart interactive educational system which integrates with the smart watch and smartphone app that can create more interactions between teachers and school students in class while providing more innovative learning possibility. In addition, it can also inspire school students and increase their concentration in the classroom. The system leveraged machine learning technology and data analysis based on the interaction results, physiological information and study performance of the students in real-time. This made teachers easily adjust the teaching process and enhance the learning efficiency of students. By field trials, we have verified that this system can potentially enhance teaching and learning process for educators and school students. In addition, it also can predict the school students’ learning scores with a very small difference. In the future work, we will keep studying on the creative design of interactive education systems to further improve the learning process for both educators and students.
